# Social capital and health: Does egalitarianism matter? A literature review

**DOI:** 10.1186/1475-9276-5-3

**Published:** 2006-04-05

**Authors:** M Kamrul Islam, Juan Merlo, Ichiro Kawachi, Martin Lindström, Ulf-G Gerdtham

**Affiliations:** 1 Department of Clinical Sciences, Lund University, Malmö University Hospital, SE-205 02 Malmö, Sweden; 2 Department of Society, Human Development and Health and the Harvard Center for Society and Health, Harvard School of Public Health, Boston, MA, USA

## Abstract

The aim of the paper is to critically review the notion of social capital and review empirical literature on the association between social capital and health across countries. The methodology used for the review includes a systematic search on electronic databases for peer-reviewed published literature. We categorize studies according to level of analysis (single and multilevel) and examine whether studies reveal a significant health impact of individual and area level social capital. We compare the study conclusions according to the country's degrees of economic egalitarianism. Regardless of study design, our findings indicate that a positive association (fixed effect) exists between social capital and better health irrespective of countries degree of egalitarianism. However, we find that the between-area variance (random effect) in health tends to be lower in more egalitarian countries than in less egalitarian countries. Our tentative conclusion is that an association between social capital and health at the individual level is robust with respect to the degree of egalitarianism within a country. Area level or contextual social capital may be less salient in egalitarian countries in explaining health differences across places.

## Introduction

The use of social factors to explain community health status is not a new phenomenon. Since Durkheim's classic work on suicide, the importance of social integration and social capital has been recognised for population well-being [1]. Nonetheless, the notion of 'social capital' has attracted wide-ranging attention in the social sciences and public health literature over the last decade [e.g., [2-10]]. In the public health arena, the concept attracted attention following the work of James Coleman and Robert Putnam [11,12]. A growing body of empirical research has been conducted on the links between individual (micro) and area (macro/meso) level social capital and population health [e.g. [13-16]].

Social capital is a multifaceted phenomenon [2]. Among other views, social capital can be considered as a by-product of social relationships resulting from reciprocal exchanges between members involved in social associations or networks and can be recognized as a public good that generates positive externalities facilitating cooperation for the achievement of common goals [17]. It is thought that social capital may generate material/market and non-material/non-market returns to the individual. Material return may include higher wage, better employment prospects or reduced transaction costs, while non-market returns may include improvements in the quality of the individual's relationships and improvements in health or even happiness [3]. It is also hypothesised that there are both direct and indirect returns on the production and accumulation of health and social capital. Direct returns stem from the fact that both health and social capital enhance individual welfare, while indirect returns come about as a result of the observation that health capital increases the amount of productive time, and social capital improves the efficiency of the production technology used for producing health capital [18].

Whilst 'egalitarianism' is a multifaceted notion in social and political thought, common forms of egalitarianism include economic egalitarianism, moral egalitarianism, legal egalitarianism, democratic egalitarianism, political egalitarianism, gender egalitarianism and opportunity egalitarianism [19]. In modern democratic societies, the term "egalitarian" is often used to refer to a situation that favors (for any of a wide range of reasons), a greater degree of equality of income and wealth across a population. Economic egalitarianism, (popular with liberals throughout much of the 20th Century), has given way to a concern not that everyone be strictly equal in material possession, but rather that everyone be equal in having enough material goods to effectively fulfill his or her native human capacities [19].

In the present paper the notion of egalitarianism is considered from material aspects and operationalized on the basis of a country's overall level of income inequality and total public social expenditure as a percentage of GDP. In a recent Luxembourg Income Study (LIS) report, Mahler & Jesuit [20] note that the Gini-index of private sector income inequality in the reputed egalitarian countries such as Sweden (44.1) and the Netherlands (45.8) are almost at the same level as in the USA (44.7) or the UK (47.5). However, the authors point out that the egalitarian distribution of disposable income (post-government income) through government transfers and taxes in Sweden and the Netherlands make the difference with the USA or the UK. Therefore, as a marker of income inequality we consider Gini-coefficients based on disposable income rather than private income. Despite this clarification, there may still remain some difficulties in classifying countries by their degree of egalitarianism. However, for the sake of simplicity we consider a country with a comparatively lower Gini-coefficient based on disposable income and a higher share of public social expenditure to be more egalitarian than a country with the reverse case. Based on country-level Gini-coefficients (based on disposable income) and share of public social expenditure in percentage of GDP, we classified countries as 'egalitarian', 'moderately egalitarian' and 'not egalitarian' as provided in Table 1[21-23]. Still, there may remain some cautions. Due to the fact that the UK is a country with a relatively higher Gini-coefficient and higher public social expenditure compared with (in our classification) moderately egalitarian countries, its position may be seen as tentative in this study. Although it may be seen as somewhat arbitrary to categorize countries as egalitarian or not, for the sake of parsimony we define Nordic countries (namely, Finland, Norway and Sweden), Germany and the Netherlands as relatively more egalitarian compared to other countries.

**Table 1:** Classification of countries by income distribution and public social expenditure, and summary of results

**Country**^¥^	**Gini coefficient**^†^	**Public social expenditure in % of GDP^Φ^**	**Single level study**				**Multilevel study**		
			
			Total number of studies	Strong association	Weak association	No association	Total number of studies	Fixed effects results	Random effects results
**Egalitarian**			**8**	**6**	**2**	**0**	**2**	**2**	
Finland	26.1	29.00	3	2	1	0	No study	-	-
Norway	26.1	25.31	1	0	1	0	No study	-	-
Sweden	24.3	32.08	3	3	0	0	1	Significant association	ICC = 0.0%
Netherlands	25.1	25.20	No study	-	-	-	1	Significant association	ICC not reported
Germany	27.7	26.70	1	1	0	0	No study	-	-
**Moderately egalitarian**			**10**	**6**	**3**	**1**	**2**	**2**	
Australia	30.5	16.99	3	1	2	0	No study	-	--
Canada	30.1	19.28	3	1	1	1	1	Significant association	ICC = 2.1%
Ireland	30.4	17.52	1	1	0	0	No study	-	-
Hungary	29.3	20.31	2	2	0	0	No study	-	-
UK	32.6	22.06	1	1	0	0	1	No conclusive evidence	ICC not reported
**Not egalitarian**			**8**	**7**	**0**	**1**	**7**	**7**	
Russia^£^	45.0	17.60	2	2	0	0	No study	-	-
USA	35.7	14.77	6	5	0	1	7	All studies show significant association	One study report ICC = 7.51%

Note:^¥^There were few studies not based on a single country but considered a group of countries (e.g. 19 OECD countries or 49 different countries from all over the world). These studies used country-level social capital measures. Therefore, we did not include these in Table 1 as these studies contain countries with all three categorization of egalitarianism. Within this cross-country category two single level studies used independent sample (for Norway and Germany) and one multilevel study based on the Netherlands. In this table we considered them as a single country study and reported accordingly.

^† ^Disposable income used for every country other than the UK (for most recent years around 2000). For the UK, household expenditure was used in estimating Gini-coefficient [See, footnote 12 of 21]. These Gini-coefficients are also comparable with the Luxembourg Income Study [See 20, Table 1].

^Φ ^OECD (2004), Social expenditure data base (SOCX, )[21]. Numbers are average value of the years 1990–2001 except for Hungary and Russia. ^£ ^For Russia, Gini-coefficient is for 1998 and Social expenditure information was available for the year 1994 [23, see, Table 9-1].

Why would a more egalitarian distribution of income and wealth matter to social capital and its impact on health? As a complementary question, one could inquire whether egalitarianism makes any difference in the formation of social capital in a society. In trying to respond to this query researchers have been puzzled by an apparently paradoxical coexistence of a greater stock of social capital and an egalitarian distribution of income and wealth through extensive welfare-state arrangements such as seen in the Scandinavian countries [24]. For instance, Alan Wolfe argued that in societies where citizens are protected "from cradle to grave" by the state, civil society and norms of reciprocity or social capital are "crowded out" [[25], p.142, also cited in [24]]. However, in reality this is not the case. Empirical research shows that in contrast to the trends described in the USA [12] there is little evidence of a decline in social capital in Sweden. Rather, in terms of trust, associational membership and informal interaction, social capital seems to have been maintained at comparatively high levels in Scandinavia [26]. For instance, the World Values Surveys in 1981, 1991–92 and 1995–96 show that 60% of the population in Sweden (second highest, 61% in Norway) agree with the statement that 'most other people can be trusted'; the percentage for the USA is 49 [27]. Recently, Kumlin and Rothstein tried to resolve this paradox empirically using Swedish data. Their findings indicate that welfare-state institutions have a capacity for both making and breaking social capital in Sweden [24]. They show that contacts with universal welfare-state institutions tend to increase social trust; by contrast, means-tested programmes tend to diminish trust.

The next issue to resolve is whether greater (lower) intensity of material egalitarianism can modify the health impact of social capital in a society. It is documented that an egalitarian distribution of wealth and income appears to imply a more cohesive, harmonious society and the level of income inequality may affect both a society's cohesiveness and its members' health [28]. By examining the relationships between income inequality, social capital and health, Wilkinson has argued that the social environment is more cohesive in more egalitarian societies; the members of these societies tend to be less violent, less prone to commit homicide, less hostile to one another, and trust other members more [29,30]. Using data from 39 US states, Kawachi and colleagues demonstrated that income inequality is positively associated with disinvestment in social capital, which is in turn linked with increased mortality [17]. Kawachi *et al. *have also shown a strong correlation between trust and income inequality in the United States, while income distribution in states was linked to variations in mortality and, indeed, death from specific causes such as heart disease, cancer and homicide [17].

Within a defined economic and political system (such as a country), the effect of different forms of social capital on health may also be conditioned by the country's intensity of economic egalitarianism. More specifically, it is arguable whether findings from countries such as the USA are generalizable to other countries which are relatively more egalitarian. Besides, unlike the conclusions of most research conducted in the USA, it has been observed that in Canada and Sweden there are no relationships between income inequality and mortality, neither at the level of provinces nor in metropolitan areas [31]. In the Swedish context, Gerdtham and Johannesson examined whether mortality is related to individual income, mean community income, and community income inequality [32]. They concluded that municipality level income inequality had no effect on mortality. At the county level, they found some indications of an association between income inequality and mortality; however, the counties which were more unequal had *lower *mortality.

Different public social institutions within welfare-states and more equitable distribution of income and wealth in the countries may modify the health impact of social capital or the effect of different forms of social capital (e.g. individual level and area level). Area level social capital (either derived from individual responses or contextual in itself) may not be relevant or important to public health if the societal level of equity or public safety net provision ensures resources for the individual. Given societal variations in economic egalitarianism, it is interesting to consider whether the effect of social capital on health varies among geographical areas according to the degree of equity and safety net provision within countries as well.

The aim of this paper is three-fold. Firstly, we critically review the origins and different forms and dimensions of social capital as it has been operationalized in the empirical literature. Secondly, we systematically review the empirical studies that have examined the health impact of individual and area level social capital for different countries by surveying both single level and multilevel studies. Thirdly, we explore some analytical and interpretational issues that may be pertinent when assessing the health impact of area level social capital. In section 4 we interpret our review results according to both single and multilevel studies and also by country, as well as by fixed and random effect results from multilevel studies. The paper ends with a discussion and conclusions along with some suggestions on directions for future research.

## Origins and definitions of social capital

(This section is mainly based on Portes [33] and Woolcock [34]).

In the literature there is no consensus on the intellectual origins or who first implicitly or explicitly introduced the notion of 'social capital'. Different authors with diverse background trace the idea from different intellectual origins. In particular, American sociologist A. Portes argues that the idea is imported from the 19^th^-century foundations of sociology and he recognizes that the concept is implicit in the works of Emile Durkheim and Karl Marx [33,35]. He further claims that Pierre Bourdieu [36] was the first to systematically and explicitly analyze the notion of social capital in the present sense. Bourdieu defines social capital as "the aggregate of the actual or potential resources which are linked to possession of a durable network of more or less institutionalized relationships of mutual acquaintance or recognition" [[37], p.248].

After reviewing the intellectual history of social capital, Woolcock notes that though employed for different purposes, the word 'social capital' was used by two major proponents of economic sciences, Marshall and Hicks, 'to distinguish between temporary and permanent stocks of physical capital' [cited in [34], p 159]. He also acknowledges the works of David Hume and Edmund Burke as intellectual origins of the notion [[34] & also cited in [35]]. Refereeing to Swedberg [38], Woolcock further claims "the Durkheimian, Weberian and Marxist traditions within classical sociology were all heavily influenced by the economic debates and issues of that period, and much of what we now refer to as 'social capital' lay at heart of these concerns" [[34] p.160]. As an explicit pioneer of the notion, Woolcock gives credit to Lyda Hanifan (in discussing rural school community centers, see [39,40] – also cited in [34], endnote 13]. Hanifan uses the term to illustrate "those tangible substances [that] count for most in the daily lives of people" ([40] p. 130). However, both Putnam [41] and Woolcock [34] refer to Jane Jacobs [42] for the first use of the term 'social capital' in its contemporary sense.

In a recent review work, Durlauf & Fafchamps [43] assert that the concept is first introduced into modern social science research by economist Glen Loury [44] who defined social capital as "naturally occurring social relationships among persons which promote or assist the acquisition of skills and traits valued in the marketplace an asset which may be as significant as financial bequests in accounting for the maintenance of inequality of our society" [[44], p.100].

Whilst it is not unambiguous who first implicitly or explicitly introduced the notion of social capital in its present sense, it is however clear that after Loury and Bourdieu, the concept is further developed, modified and disseminated in the diverse disciplines by the works of Coleman [11], Putnam *et al. *[45] and Portes [33]. The definitions given by these proponents of the notion are cited as follows:

**Coleman: **"consist of some aspect of social structure and they facilitate certain actions of individuals who are within the structure" [[11], p.302].

**Putnam et al: **"refers to features of social organization, such as trust, norms and networks that can improve the efficiency of society by facilitating coordinated actions" [[45], p.167].

**Portes: **"refers to the capacity of individuals to command scare resources by virtue of their membership in networks or broader social structure" [[33], p.12].

Although overlapping to some extent, four main theoretical ingredients can be identified in the definition of social capital: social trust/reciprocity, collective efficacy, participation in voluntary organizations and social integration for mutual benefit [9]. After surveying different empirical studies and definitions of social capital, Durlauf & Fafchamps distinguish three main basic ideas:"(a) social capital generates positive externalities for members of a group; (b) these externalities are achieved thanks to shared trust, norms and values and their effects on expectations and behavior; and (c) shared trust, norms and values arise from informal forms of organizations based on social network and association. The study of social capital is that of network-based process that generate beneficial outcomes through norms and trust". [[43], p.5].

### The forms and dimensions of social capital

**Figure 1:**
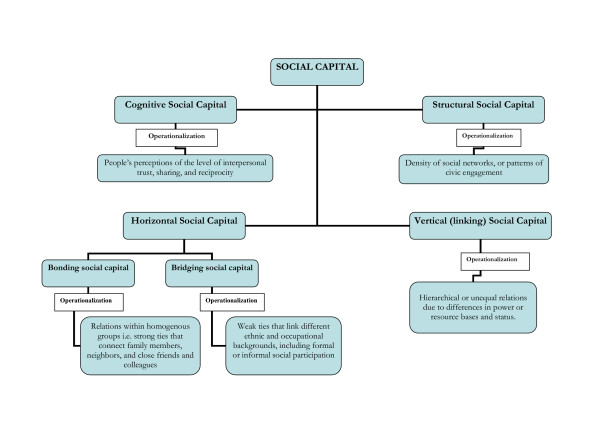
Forms and dimensions of social capital with operationalization of the notion in empirical studies.

As seen in Figure 1, social capital can be broken down into cognitive and structural components [46-48]. Cognitive social capital includes norms, values, attitudes and beliefs. Structural components of social capital refer to externally observable aspects of social organization, such as the density of social networks, or patterns of civic engagement. The structural components of social capital are contextual in its nature. Structural and cognitive social capital are complementary. The cognitive component assesses people's perceptions of the level of interpersonal trust, sharing, and reciprocity. The structural component of social capital examines the extent and intensity of associational links and activity in society such as measures of informal sociability, density of civic associations, and indicators of civic engagement [47]. In relation to health, cognitive social capital (predominantly captured at the micro level) is believed to shape behavioral norms, through control of risk behavior, provision of mutual aid and support, and informal means of informational exchange [49]. Structural social capital at the macro level is shaped by institutions, policies, and culture. Different forms and dimensions of social capital along with their operationalization are also specified in figure 1.

As noticed in Figure 1, two distinct types of social capital are recognized – *horizontal*, reflecting ties that exist among individuals or groups of equals or near-equals, and *vertical *(also referred to as *linking *social capital), stemming from hierarchical or unequal relations due to differences in power or resource bases and status [50]. Additional distinctions have been drawn within horizontal social capital, viz., "bonding" social capital (also called localized social capital) and "bridging" social capital [12,51]. Bonding social capital refers to the relations within homogenous groups. In other words, these are the strong ties that connect family members, neighbors, and close friends and colleagues. By contrast, bridging social capital is heterogeneous by definition. The weak ties that link those of different ethnic and occupational backgrounds form "bridging" social capital, including formal or informal social interactions [12,50-53].

Bonding relationships act as the primary means for the transmission of behavioral norms to family members and friends. Bonding social capital is important for establishing and favoring healthy norms, controlling abnormal social behavior and for generating mutual aid, and protecting the vulnerable [49]. By contrast, bridging social capital is important to the success of civil society and it is also recognised as an important source of other benefits for individuals, communities, and societies. It offers members of the society opportunities for participation in heterogeneous groups of people from diverse social classes and opens channels to voice concern in favor of those who may have very little opportunity to reach more formal avenues in order to affect societal changes, e.g. change in public welfare-oriented policies [49]. Virtually no studies have explicitly measured and tested the bridging form of social capital and its relationship to health. Theoretically, bridging social capital may be associated with better health because it enables disadvantaged groups to access material resources through connections to socially advantaged groups. However, in one of the few published studies to measure bridging social capital, Mitchell and LaGory found *bonding *social capital was associated with *worse *mental health in a disadvantaged black community in the USA, whereas *bridging *social capital was associated with better mental health [54]. Bridging social capital may also be critical for the prevention of inter-ethnic and religious conflict and violence [55].

### Critiques of social capital

A common criticism of the concept of social capital concerns measurement. The meaning of the concept continues to be contested, and consensus about its measurement remains elusive. Baum finds that the current concepts of social capital are "vague, slippery, and poorly specified, and in danger of 'meaning all things to all people' on both the right and left of the political spectrum" [56]. Gillies depicts social capital as "a descriptive construct rather than an explanatory theory" [[57] & cited in [58]]. Like other social scientists, economists have also criticized the definitions of social capital, pointing out the vagueness and inconsistency of various definitions [which may be found in e.g. [2,43,59] &[60]].

There are also some ambiguities concerning where exactly social capital resides. One important distinction is between social capital as an individual attribute and social capital as a collective characteristic [10,35]. Almost all approaches to the measurement (or conceptualization) of social capital confuse the differences between social capital as a social resource, as a social product, or as an individual response [35]. With the exception of Portes, this problem is inherent in the work of all major proponents of the concept of social capital. By contrast, Portes has clearly described social capital as an individual attribute [33,35].

Moreover, the operationalization and measurement of social capital remains a challenging task to researchers. From the perspective of empirical research, quantifying social capital involves identifying observable variables that can be used as proxies for social capital [33]. Additionally, it is also recognized that indicators such as 'norms' and 'shared values' are notoriously difficult to measure [43]. Furthermore, indicators of social capital are not routinely available on administrative data sets (such as the government census). Even when special surveys have been collected to measure social capital, the relevant spatial scale for operationalizing the concept (the neighborhood versus the state, or even entire societies) presents an analytical dilemma [9,35,61,62]. The Modifiable Area Unit Problem (MAUP) has been acknowledged as a potentially worrying feature of aggregated data. MAUP is a result of the imposition of artificial units of spatial aggregation on a continuous geographical phenomenon, resulting in the construction of an artificial spatial pattern [63,64]. MAUP is not unique to the analysis of social capital and has also been found in the analysis of area-based socioeconomic and epidemiological data [65,66].

Social capital is not a panacea for population health problems and it may not always generate better health outcomes. Researchers in social capital have been criticized for failing to consider such negative outcomes. Social capital can sometimes facilitate negative or perverse consequences. Portes acknowledges four instances of negative consequences: exclusion of outsiders from resources controlled by network members, excess claims made on successful members by free-riding fellow members, restrictions on individual freedoms (particularly in closely bonded networks), and the downward leveling of norms, which may block members of historically oppressed groups from participation in mainstream society [33]. Similarly, Baum recognizes that interconnection or close-knit association may not necessarily be 'healthy', particularly for outsiders [56]. Muntaner *et al. *also point out that strong associations among individuals may both increase and decrease the risk of certain health outcomes [67]. For example, strong friendship networks of peers may increase the risks of smoking, drinking, or use of illicit drugs, while in different circumstances these same sorts of connections may decrease the risk of suicide. In the Swedish context, using cross-sectional survey data, Lundborg examines school class-based peer effects in binge drinking, smoking and illicit drug use and finds significant positive peer effects for all three activities [68].

Finally, the literature on social capital and health has also been criticized by neo-materialists who emphasize the importance of political regimes, ideology, and institutions for good health both at the population and individual level [69]. The neo-materialists have even accused social capital theorists of "blaming the community" for their problems, such as poor health outcomes [69,70]. Navarro criticizes the social capital literature for exaggerating its importance for health. He also points out that class relations are absent from social epidemiology and public health research – class relations may be a more important determinant of population health than social capital [71].

## Methods

### Search strategy and inclusion criteria

The methodology used was a systematic search on electronic databases for published literature. A detailed search of the databases was conducted for articles published between January 1^st ^1995 and June 15^th^, 2005: MEDLINE (via Pub Med), Sociological Abstracts (via CSA), EconLit (via CSA), and International Bibliography of the Social Sciences, IBSS, (via CSA). The review used the following key words: "social capital" AND "health" OR "mortality" OR "self-rated health" OR "morbidity".

We mainly included the social capital studies that consider direct health status measures and mortality as outcome variable(s) and studies that used a quantitative methodology and were published in peer reviewed journals. Once the searches were completed, the title, key words, and abstracts were reviewed for final selection. Unpublished data were excluded from these analyses, as well as articles published in languages other than English. In total, over 653 articles were initially identified as potentially fitting the selection criteria (233 from Medline and the rest from other data bases, i.e. EconLit (103), Sociological Abstracts (229) and IBSS (88). From the initial searches, articles were excluded where the title and abstract made it clear that the paper did not fulfill the inclusion criteria. Collectively, these search strategies resulted in a total of 42 articles fitting the study inclusion criteria.

### Classification of studies

Macinko and Starfield state that social capital can be analyzed at four different levels: the macro level (countries, regions and counties), the meso level (neighborhoods), the micro level (social networks of individuals) as well as the individual attitudinal/psychological level (trust) [35]. Furthermore, depending on analytical approach and study design, health impact of social capital studies can be classified by the level of analysis and unit of analysis. With regard to the level, a study may be a single level or a multilevel study. To differentiate between *single level *and *multilevel *studies, we indicate whether studies use an analytical approach that explicitly recognizes a hierarchical structure for the data.

Depending on the study design (unit of analysis), single level studies can be further distinguished as *individual level studies *and *ecological studies*. In individual studies, the unit of observation and analysis is the subjects (all variables, e.g. response variable – health status and predictor – social capital, are gathered as individual attributes). In ecological studies the unit of analysis is a group of individuals who are clustered together according to geo-demographic, socioeconomic, or other criteria (e.g. state/municipality/neighborhood as unit of analysis) [72]. In the ecological studies both health status and social capital are examined at the aggregate level.

Multilevel analysis studies are concerned with analyzing data with a nested structure. The main reason for employing multilevel statistical analysis is because we are interested in explicitly modeling the contextual heterogeneity. In multilevel analysis, the term 'contextual effects' is generally used to refer to the effects of variables (e.g. social capital) at a higher level (state/community/neighborhood) on outcomes defined at a lower level (e.g. individual level health status)[73]. In multilevel analysis, one may also control for individual level social capital, when analyzing area level social capital variables or 'contextual social capital'. Also, the term 'contextual social capital' refer occasionally to the effects of area level social capital variables and may be classified as a '*derived variable*' – summarizing specific characteristics of individuals within the group or area (e.g. overall civic participation or level of trust) – and '*integral variable*'- no individual level analogues but rather a group or area level construct (e.g. number of voluntary organizations/political parties in an area, crime rate etc) [73].

To avoid the confusion of trying to identify the different kinds of social capital used in the reviewed studies, we categorize social capital into three groups, irrespective of different study design: individual level social capital, 'aggregated' level social capital (individual social capital but aggregated to area level or *derived *as an area level social capital) and contextual social capital (*integral *social capital variable).

## Results

A total of 42 papers were reviewed. These papers come mostly from OECD countries, representing a total of 30 single-level studies (17 individual level and 13 ecological studies) and 12 multilevel studies. These comprised of 13 papers (6 multilevel studies) using US data, 4 studies (1 multilevel) from Canada, 4 from eastern Europe (no multilevel study), 4 from western Europe (2 multilevel studies), 7 from Scandinavia (1 multilevel), 3 from Australia (no multilevel study), and 7 cross-country studies (1 multilevel). A concise summary of the studies on the associations between social capital and health status (mortality or self-rated health/health) by different countries is presented in Table 1. Tables 2a and 2b summarize the sources and characteristics of the studies, i.e. (a) study design (unit of analysis- whether ecological or individual based analysis, data etc), (b) conceptualization and operationalization of the social capital variable (how social capital is defined and measured and kind of measurement), and (c) outcome variable(s) (which response variable(s) was considered for the studies) along with (d) their main findings. In the last column of Table 2a, countries are classified according to their degree of egalitarianism.

**Table 2:** Empirical evidence by types of studies and countries: Single level studies

**Study (Reference)**	**Study design/Data**	**Social-capital measures**	**Type of social capital**	**Outcomes**	**Findings**	**Degree of egalitarianism**
**Studies in North America (USA & Canada)**						
**Kawachi, Kennedy, Locher & Prothrow-Stith, 1997 [17]**	A cross-sectional ecological study based on data from 39 US states. Socioeconomic data were obtained from 1990 US Census Population and Housing Summary Tape File 3A.	Using General Social Survey (GSS) responses on social trust, perceived lack of fairness, perceived helpfulness of others and memberships in groups- taking each one separately.	'Aggregated' social capital (individual level responses aggregated to the US state level).	Income inequality and mortality in US states.	Each of the four measures was found to be positively associated with mortality.	Not egalitarian
**Wilkinson, Kawachi & Kennedy, 1998 [74]**	A cross-sectional study data used from the US General Social Surveys (1986–90) and National Center for Health Statistics (1981–1991).	Social mistrust was used as indicator of social capital.	'Aggregated' social capital (individual level responses aggregated to US state level)	Mortality and violent crime rate in 39 US states.	Social mistrust closely related with mortality and violent crime rates.	Not egalitarian
**Lochner, Kawachi, Brennan & Buka, 2003 [75]**	A cross-sectional study design for persons 45–64 years, indicators of neighborhood social capital were obtained from the 1995 Community Survey of the Project on Human Development in Chicago Neighborhoods.	Measured by reciprocity, trust, and civic participation.	'Aggregated' social capital (individual level responses aggregated to neighborhood level)	Mortality rates in 342 Chicago neighborhoods, USA.	Higher levels of neighborhood social capital were associated with lower neighbourhood death rates for total mortality as well as death from heart disease and "other" causes for death.	Not egalitarian
**Holtgrave & Crosby, 2003 [76]**	An ecological state level study where data used from Putnum, 2001 [US General Social Surveys (1974–1994); DDB Needham archive (1975–1998); Roper Social and Political Trends archive (1974–1997)] Youth Risk Behavior Surveillance Survey (1999).	Includes 14 variables that span the domains of community organizational life, involvement in public volunteerism, informal sociability and social trust and called this "comprehensive social capital index".	Combination of 'aggregated' social capital (individual level responses aggregated to the US state level) and contextual social capital	Case rates of gonorrhoea, syphilis, Chlamydia and AIDS in 48 US States.	Social capital index inversely associated with gonorrhoea, syphilis, Chlamydia and AIDS case rates.	Not egalitarian
**Milyo & Mellor, 2003 [77]**	An ecological analysis based on state-level data came from several publicly available sources. Crude and age-adjusted mortality rates for 1990 are obtained from the US Centers for Disease Control. The Gini-coefficient for family income and percent of persons below the federal poverty line are come from the US Bureau of Labor Statistics. Other than social capital all covariates were obtained from the US Census Bureau.	Social capital was measured by Robert Putnam's index of state social capital and by an index of social mistrust derived from responses to the General Social Survey, following the method described in [17]	Combination of 'aggregated' social capital (individual level responses aggregated to US state level) and contextual social capital	Age-adjusted mortality rates, defined as deaths per 100,000 in 1990.	The study did not find significant association between mortality and either minority racial concentration, or social capital. Authors conclude that different age-adjustment methods can cause a change in the sign or statistical significance of the association between mortality and other socioeconomic factors.	Not egalitarian
**Holtgrave & Crosby, 2004 [78]**	An ecological state level study data came from Putnum, 2001 [US General Social Surveys (1974–1994); DDB Needham archive (1975–1998); Roper Social and Political Trends archive (1974–1997)] Youth Risk Behavior Surveillance Survey (1999).	Includes 14 variables that span the domains of community organizational life, involvement in public volunteerism, informal sociability and social trust and called this "comprehensive social capital index".	Combination of 'aggregated' (individual level responses aggregated to the US state level) and contextual social capital	Tuberculosis case rates in 48 US States.	Social capital index inversely associated with tuberculosis case rates.	Not egalitarian
**Veenstra, 2000 [79]**	A cross-sectional study, data used from a survey administered to randomly selected citizens within randomly selected households from eight health districts of Saskatchewan in 1997.	Constructing different indices for overall civic participation, trust in government, trust in neighbours, trust in people from respondents' communities, trust in people from respondents' part of Saskatchewan, and trust people in general.	Individual level social capital	Self-rated health within Saskatchewan, Canada.	Social capital was not significantly related to self-rated health among the general population but positive impact among the elderly.	Moderately egalitarian
**Veenstra, 2002 [80]**	A cross-sectional study, data came from the Canadian National Population Health Survey, considered 30 health districts in Saskatchewan, Canada.	The social capital index incorporated associational density and civic participation.	Individual level social capital	Mortality rates; low birth weight rate; proportion of residents receiving mental health services etc.	Except low birth weight rate social capital was inversely and weakly related to age-adjusted mortality rates, and other outcomes.	Moderately egalitarian
**Veenstra *et al*. 2005 [81]**	A cross-sectional study based on an individual level data from a telephone survey of a random sample of adults (N = 1504) neighborhood of residence in the city of Hamilton, Canada The survey contained a range of questions designed to capture participation in social and community networks, health status and behaviors, use and access of health services and socio-demographic factors was administered to respondents between November 2001 and April 2002.	Social capital measured by constructing an index focusing specifically on breadth and depth of involvement in voluntary associations where respondents were asked if they belonged to an association and assessed its type, i.e., religious, cultural/historic, community, social services/health, sports/athletics, pastimes/social/artistic, professional or political assessing degree of involvement in the association. The minimum score on this index was zero (no groups were mentioned) and the maximum was 6.0.	Individual level social capital	Various measures of individual health, such as- self-rated health, chronic conditions, emotional distress and body-mass index.	Overall involvement in voluntary associations was significantly related to emotional distress and almost significantly related to self-rated health before and after controlling for age, gender and neighbourhood of residence. More participation in voluntary associations apparently had a positive association with these measures of health.	Moderately egalitarian
**Studies in eastern Europe**						
**Kennedy, Kawachi & Brained, 1998 [8]**	Cross-sectional, ecological analysis across 40 regions of Russia. The study based on a stratified random sample of the population, survey conducted by the All-Russian Center for public opinion in April through June, 1994	Indicators used for social capital such as civic engagement, and trust in government and social cohesion (divorce rate, per capita crime rate, conflicts in workplace) ;	Both 'aggregated' social capital (individual level responses aggregated to the region of Russia) and contextual social capital	Mortality in Russia.	Social capital and cohesion indicators were strongly linked with age-adjusted mortality for both sexes.	Not egalitarian
**Rose, 2000 [82]**	Based on a nation-wide cross-sectional survey (New Russia Barometer Survey) conducted in March-April, 1998.	Sense of self-efficacy, trust of others, inclusion or exclusion from formal and informal networks, social support and social integration,	Individual level social capital	Self-reported physical and emotional health in Russia.	Both human and social capital were associated with improved self-reported health.	Not egalitarian
**Skrabski, Kopp & Kawachi, 2003 [83]**	A cross-sectional ecological study based on The Hungarostudy II, a national survey representing the Hungarian population over the age of 16 conducted for the 20 counties in 1995.	Measured by three indicators: lack of social trust, reciprocity between citizens and help received from civil organization	'Aggregated' social capital (Individual level responses aggregated to the counties of Hungary)	Gender specific mortality rates for the middle aged population in the 20 counties of Hungary Life satisfaction, suicide rates for 50 countries.	All of the social capital variables were significantly linked with middle age mortality.	Moderately egalitarian
**Skrabski, Kopp & Kawachi, 2004 [84]**	A cross-sectional ecological study based on The 'Hungarostudy' survey 2002 and Hungarian Central Statistical Office (1996–2000).	Social trust, organization membership and reciprocity were used as indicators of social capital.	'Aggregated' social capital (Individual level responses aggregated to the sub region of Hungary)	Gender specific all cause specific mortality rates in the 150 sub-regions of Hungry.	Social distrust positively and other two indicators inversely and significantly associated with all cause mortality rates.	Moderately egalitarian
**Studies in western Europe**						
**McCulloch, 2001 [85]**	A representative cross-sectional study based on the British Household Panel Study for the years 1998 and 1999.	Summed individual responses to eight questions about different community problems and classified them into low, medium, high and very high levels of social disorganization.	Individual level social capital	Examined psychiatric morbidity using the 12 item general health questionnaire for a representative cross section of British households.	People in the lowest categories of social capital had increased risk of psychiatric morbidity.	Moderately egalitarian
**Kelleher, Timoney, S Friel & McKeown, 2002 [86]**	A cross-sectional ecological study employed three data sources namely,1996 census data from the Central Statistics Office, 1997 general election first preference voting data in all 41 constituencies were aggregated to county level and the National survey on lifestyles, attitudes and nutrition (SLAN) data. The study comprised adults over 18 years sampled by post using the electoral register from 273 representative district electoral divisions.	Party political affiliation and general election voting pattern was used to measure vertical social capital.	Contextual social capital	Standardised mortality ratios (SMR) and selected reported measures of health status, lifestyle in Ireland.	There was no significant relation between SMR and voting pattern for the two main political parties but a significant relation with left wing voting. There was a positive significant relation between left wing voting and dissatisfaction with health and rate of smoking.	Moderately egalitarian
**Studies in Scandinavia**						
**Hyyppä & Mäki, 2001b [88]**	An individual level cross sectional study based on a randomly selected samples of Finnish-speakers (N = 1,000), and Swedish-speakers (N = 1,000), representing all adults living in bilingual Ostrobothnian municipalities. 75,000 Finnish speakers and 78,000 Swedish-speakers from national registers of the Social Insurance Institution of Finland (KELA) and stratified by gender, age, and municipality.	Social capital operationalized by social ties and integrity operationalized by asking four questions on friendship, voluntary neighbourhood assistance, reciprocal civic trust, and civic engagement.	Individual level social capital	Self-rated good health.	Swedish speaking community appeared to hold a better stock of social capital than Finnish speaking counterparts which were significantly and positively associated with good self-rated health.	Egalitarian
**Hyyppä & Mäki, 2003 [89]**	An individual level cross- sectional study used randomly selected samples of Finnish-speakers (N = 1,000), and Swedish-speakers (N = 1,000), representing all adults living in bilingual Ostrobothnian municipalities. 75,000 Finnish speakers and 78,000 Swedish-speakers from national registers of the Social Insurance Institution of Finland (KELA) and stratified by gender, age, and municipality.	Active participation in voluntary associations, friendship ties, religious involvement and hobby club activity and trust were used as measures of social capital.	Individual level social capital	Self-rated good health.	Active participation in voluntary associations, friendship ties and trust associated with self-rated good health.	Egalitarian
**Liukkonen, Virtanen, Kivimäki, Pentti & Vahtera, 2004 [90]**	The study based on a prospective cohort of 6028 public sector employees in Finland. In the 10-Town Study sent out a questionnaire to all full-time permanent employees who were at work at the time of survey in the eight towns participating in the study in 1997.	Employment security and social support two indicators reflecting employment type and co-worker support, and combined them into a variable indicating the amount of 'social job capital'.	Individual level social capital	A 5-point scale of self-rated health and Psychological distress was measured by the 12-item version of the General Health Questionnaire.	The results indicated that a low level of 'social job capital' is associated with poor health only in the age-adjusted model in women. However, after accounting for baseline health differences and other background variables, the significant associations were disappeared both in women and in men.	Egalitarian
**Bolin, Lindgren, Lindstrom & Nystedt, 2003 [18]**	The study employed a set of individual panel data based on the Swedish survey of living conditions (ULF). The panel consisting of about 3800 individuals, for the years 1980/81, 1988/89 and 1996/97.	As an indicator of social capital the authors considered whether individual had a close friend outside his or her household.	Individual level social capital	Self-rated health in Sweden.	Social capital had a positive effect on self-assessed health.	Egalitarian
**Sundquist, Lindström, Malmström, Johansson & Sundquist, 2004 [91]**	A cross-sectional follow-up study based on data from the Swedish Annual Level-of-Living Survey (SALLS). During 1990 and 1991, 6861 women and men aged 35–74 were interviewed.	Neighbors talk often in the area, whether attended mutual activity in the neighbourhood and whether socialize with neighbors at least once every three months were used as indicators of social capital.	Individual level social capital	Coronary heart disease (CHD) morbidity and mortality in Sweden.	Persons with low social participation in the social participation index exhibited an increased risk of CHD.	Egalitarian
**Lindström, 2004 [92]**	A cross-section study, data came from the population investigated by a postal questionnaire in Scania in southern Sweden during November 1999-February 2000, the public health survey in Scania 2000. The postal questionnaire was sent to 24,922 randomly chosen persons aged 18–80 (born in 1919–1981) that were registered as living in Scania.	Social participation measured as how actively the person takes part in the activities of formal and informal groups in society during last year and Generalised trust to other people is a self-reported indicator that reflects the person's perception of generalised trust to other people With the combination of social participation and trust, four alternatives social capital levels are identified such as, high-social participation/high trust (*high social capital*), high-social participation/low trust ("*the miniaturisation of community*"), low-social participation/high trust (*traditionalism*), and low-social participation/low trust (*low-social capital*).	Individual level social capital	Self-rated health and psychological health (GHQ12) in southern Sweden.	For both sexes with low trust have significantly higher odds ratios of bad self-reported global health. The odds ratios of bad self-reported health are significantly higher in the categories high-social participation/low trust (miniaturisation of community), low-social participation/high trust (traditionalism) and low-social participation/low trust (low-social capital) compared to the high-social capital (high-social participation/high trust) category among both men and women. The highest odds ratios of bad self-reported global health are observed in the low-social capital categories in both sexes.	Egalitarian
**Studies in Australia**						
**Siahpush & Singh, 1999 [93]**	A state level ecological study and data complied from several Australian Bureau of Statistics documents for the years 1990–1996.	Five indicators of social integration namely percentage of people living alone, divorce rate, unemployment rate, proportion of people who are discouraged job-seekers and unionization rate were used as proxy of social capital.	Contextual social capital	State level six Cause-specific mortality, all cause mortality and sex specific life expectancy in Australia.	Higher levels of social capital for most of the indicators were significantly associated with mortality rates and life expectancy.	Moderately egalitarian
**Chavez, Kemp & Harris, 2004 [94]**	A household level cross-sectional survey based study originally developed as evaluation tool for neighborhood based interventions and used for two disadvantaged neighborhood in south-western Sydney, Australia.	Six common social capital components such as, neighbourhood attachment, support networks, feelings of trust and reciprocity, local engagement, personal attachment to the area, feelings about safety and pro-activity in the social context were used.	Individual level social capital	Self-reported health.	It is revealed that with the exception of feelings of trust and reciprocity, no other social capital component made significant contributions to explaining health variance and those macro-level factors such as housing conditions and employment opportunities emerged as key explanatory factors.	Moderately egalitarian
**Ziersch, Baum, MacDougall & Putland, 2005 [95]**	Data came from a broader study, the Health Development and Social Capital Project (HDSCP), undertaken in the Western suburbs of Adelaide in 1997. Two sources of data come from HDSCP through a questionnaire (n = 2400) and in-depth interviews (n = 40).	Social capital operationalized by neighbourhood connections, neighbourhood trust, reciprocity, neighbourhood safety, local civic action.	Individual level social capital	Physical and mental health as measured by SF-12 (a summary scores used as response variable) for the resident live in Adelaide, Australia	Only perceived neighborhood safety was related to physical health and neighbourhood connections and neighbourhood safety were positively associated with mental health, with those with stronger neighbourhood connections and higher levels of perceived neighbourhood safety, having better mental health.	Moderately egalitarian
**Cross-Country Studies**		
**Lynch, Davey Smith, Hillemeier, Shaw, Raghunathan & Kaplan, 2001 [96]**	A cross-sectional study data came from the World Values Survey (1990–1991); UN Human Development Report and WHO mortality data base (1991–1993).	Used social distrust, organization membership and volunteering as indicators of social capital.	'Aggregated' social capital (Individual level responses aggregated to different country level)	Life expectancy, mortality, low birth-weight and self-rated health for 16 OECD countries.	Difference between countries in levels of social capital showed weak and inconsistent associations with age-specific and cause-specific mortality rates.	NA
**Kennelly, O'Shea & Gavey, 2003 [97]**	The study used a panel data set covering three time periods and the trust data comes from the three waves of the World Values Survey: 1981–84, 1990–93 and 1995–97.	Measured by the proportion of people who say that they generally trust other people and by membership in voluntary organization.	'Aggregated' social capital (Individual level responses aggregated to different country level)	Population health in 19 countries in the OECD.	Found very little statistically significance evidence of social capital had a positive effect on population health.	NA
**Smith & Polanyi 2003 [98]**	A cross-sectional study data came from the 1995–97 World Values Survey conducted in a variety of countries including Australia, Sweden and Norway (n = 5,096).	Social capital operationalized through socially oriented norms and behaviors.	Individual level social capital	The gradient between income and self-rated health across three different welfare countries.	The study found variation in the level of social capital measures across the three different countries. Socially oriented norms were not strongly correlated with each other, or with socially oriented behaviors. And existence of socially oriented norms or behaviors did not reduce the likelihood of lower income groups reporting poor self-rated health, relative to the highest income groups.	NA
**Carlson, 2004 [99]**	An ecological cross-sectional study based on data from the World Value Survey conducted in 1995–1997 and based on data from 18 European countries and from respondents aged 18 years and over.	Measured from an individual perspective, where the individual's trust in people, confidence in the legal system or membership of organizations are investigated.	Individual level social capital	Self-rated health constructed as 'Very good' and 'good' were defined as good health and 'satisfactory', 'poor' and 'very poor' were defined as poor health.	Both economic factors and some aspects of social capital played a role in the area differences in self-rated health. Economic factors appeared to be more important. People in the countries in central and eastern Europe tended to be worse off than in western Europe, both in terms of economy and in terms of social capital.	NA
**Helliwell & Putnam, 2004 [100]**	A cross-sectional study, data came from three different sources of survey data. The first source was the World Value Survey (WVS) of the years 1980, 1991–1992 and 1995–1997, covered 49 countries, and used a three-wave panel of roughly 84,000 observations. The second data source was the Social Capital Benchmark Survey in the US includes about 29, 000 observations drawn from a national random sample supplemented by samples from many participating communities The third source was the Canadian data were drawn from two national waves and two special over-samples of a survey sponsored by the Social Sciences and Humanities Research Council of Canada and the sample used in the analysis was about 7500.	Social capital operationalized by the strength of family, neighbourhood, religious and community ties.	Individual level social capital	Life satisfaction, happiness and self assessed health status measured on the same five point scale used in all three surveys.	Social capital was strongly associated with subjective well-being through many independent channels and in several different forms. All indicators of social capital appeared independently and robustly related to happiness and life satisfaction, both directly and through their impact on health.	NA
**Pollack & Knesebeck, 2004 [101]**	A cross-national study based on Germany and the United States in the years 2000 and 2001. Data obtained by computer assistance telephone interviews (CATI) conducted in Germany (N = 682) and the United States (N = 608) with probability samples of non-institutionalized persons aged 60 and older was used.	Social capital operationalized by both norms (reciprocity and civic trust) and behaviors (participation). Participation was assessed by whether people attended a church, charity group, sports club, self-help group, or other local activity at least once a month.	Individual level social capital	Three self-reported health indicators overall health, depression (CES-D) and functional limitations.	Lack of reciprocity was associated with poorer self-rated health and depression in both countries and civic mistrust was associated with poorer self-rated health in both countries. Lack of participation was, associated with poorer self-rated health and depression in Germany, The effect of norms is stronger in the US than in Germany. Participation in community groups, however, is more strongly associated in Germany.	NA

**Table 3:** Empirical evidence by types of studies and countries: Multilevel studies

**Study (Reference)**	**Study design/Unit of analysis**	**Social-capital measures**	**Type of higher level social capital**	**Outcomes**	**Fixed effects results**	**Random effects results**
**Studies in North America(USA)**						
**Sampson, Raudenbush & Earls, 1997 [107]**	Cross sectional data came from 1995 Project on Human Development in Chicago Neighborhoods, 8782 individuals in 343 Neighbourhood clusters in Chicago. Level 1: Individual (micro) Level 2: Neighborhoods (Meso).	Collective efficacy, defined as social cohesion among neighbors combined with their willingness to intervene on behalf of the common good.	'Aggregated' social capital (individual level responses aggregated to neighborhood level).	Violent crime and homicide in Chicago, the USA.	Collective efficacy negatively associated with neighbourhood variations in violent crime and homicide.	Variance components both within neighborhoods (0.320) and between neighbourhoods (0.026) for collective efficacy estimated and ICC is 7.51%.
**Kawachi, Kennedy, & Glass, 1999 [7]**	Cross-sectional data among 167,259 respondents came from the Centers for Disease Control Behavioral Risk Factor Surveillance Surveys. Level 1: Individual (micro) Level 2: States (Macro).	Using three GSS measures of civic trust, reciprocity (helpfulness of others) and civic engagement (membership in group) and based on these indices states were characterized as high, medium and low social capital	'Aggregated' social capital (individual level responses aggregated to state level).	Self-rated health between US states.	Person living in a state with low levels of social capital had an increased probability of lower self-rated health than someone living in an area of higher social capital.	Variance component for both levels and/or ICC was not reported.
**Subramanian, Kawachi & Kennedy, 2001 [108]**	Cross-sectional data used from the 1993–94 Behavioral Risk Factor Surveillance System and the 1986–90 General Social Surveys. Level 1: Individual (micro) Level 2: States (Macro).	Operationalized as the percent of residents in each state responding that 'other people would try to take advantage of you if they could (mistrust).	'Aggregated' social capital (individual level responses aggregated to state level).	Self-rated health between US states	After controlling for income-inequality and overall income a significant effect of social capital was observed.	Variance component for both levels and/or ICC was not reported.
**Subramanian, Kim, & Kawachi, 2002 [14]**	Cross-sectional data among 21,456 individuals nested within 40 US communities included in the 2000 Social Capital Community Benchmark Survey. Level 1: Individual (micro) Level 2: States (Macro).	Perceptions of individual trust were derived by summing individual responses on (1) general interpersonal trust and (2) degrees of trustworthiness of neighbors, co workers, fellow congregants, store employees where the individual shops, and local police. At the community level, a contextual social trust variable was aggregated from individual responses to questions on interpersonal trust.	'Aggregated' social capital (individual level responses aggregated to state level).	Self-rated health between US states.	High community levels of social trust and self-rated health are positively associated, a significant cross-level interaction effect between community and individual trust also observed.	Variance component for both levels and/or ICC was not reported.
**Browning & Cagney, 2002 [109]**	Cross sectional data came from 1994 Project on Human Development in Chicago Neighborhoods, 1991–2000 Metropolitan Community Information Center-Metro Survey; 2218 individuals in 333 Neighbourhood clusters in Chicago. Level 1: Individual (micro) Level 2: Neighborhoods (Meso).	Collective efficacy such as reciprocity, density of local networking, social cohesion, informal social control used for conceptualizing social capital.	'Aggregated' social capital (individual level responses aggregated to neighborhood level).	Self-rated physical health between Chicago Neighborhoods, the USA.	Higher levels of neighbourhood collective efficacy associated with better self-rated overall health.	Variance component for both levels and/or ICC was not reported.
**Wen, Browning & Cagney, 2003 [110]**	A cross-sectional data employed from 1990 Decennial Census; the 1994–95 Project on Human Development in Chicago Neighborhoods-Community Survey and the 1991–2000 Metropolitan Chicago Information Center Metro Survey for 8782 individuals in 343 neighborhoods clusters in Chicago. Level 1: Individual (micro) Level 2: Neighborhood (Meso).	Collective efficacy such as reciprocity, density of local networking, social cohesion, informal social control used for conceptualizing social capital.	'Aggregated' social capital (individual level responses aggregated to neighborhood level).	Self-rated health in Chicago neighborhoods in the USA.	Neighbourhood social capital associated with better individual self-rated health.	Variance component for both levels and/or ICC was not reported
**Franzini & Spears, 2003 [111]**	A cross-sectional study based on Texas, USA, in 1991. Using the 1990 US census of total 61,557 heart disease deaths in Texas in 1991 recorded, 54,640 (89%) were linked to the census information by geocoding and the individual's addresses were geocoded to12,344 block-groups, 3788 tracts, and 247 counties in Texas. Level 1: Individual (micro) Level 2: Block-group level (Meso) Level 3: Tract Level (Meso) Level 4: County (Macro).	Social capital as one of the indicators of social context was operationalized by homeownership (percent of owner-occupied housing units) at the tract and county level and the crime index (defined as number of serious crimes known to police per 100,000 population) at the county level.	Contextual social capital	Premature mortality from heart disease. Years of potential life lost were computed as the 1990 life expectancy in Texas at age when death occurred.	Individual level characteristics were major predictors. Social context at the block-group, tract, and county level plays an important role in explaining years of life lost to heart disease. Block-group level wealth, tract level own group ethnic density, and county level social capital, had significant effect on years of life lost to heart disease in Texas.	Variance component for both levels and/or ICC was not reported.
**Veenstra, 2005 [112]**	A cross-sectional study data came from two original data sets, one pertaining to features of 25 communities in British Columbia, Canada and the other to characteristics of individuals living in them. Individual responses (N = 1435) collected from a mailed survey of randomly selected residents aged 18 and higher during the summer and fall of 2002. A random selection of households was drawn from the most current telephone listings using a systematic random sampling technique, and a survey questionnaire was then administered by post in a five-stage process. Level 1: Individual (micro) Level 2: Community (Meso).	Individual-level social capital was operationalized through individuals' perception about social and political trust and participation in voluntary associations. To measure attributes of communities the study determined (i) the number of public spaces per capita (sports, recreational, casual and social, cultural, religious, school and hall spaces in particular), (ii) the number of voluntary organizations per capita (sports and athletics, community, minorities, arts and culture, business, political, health and social services, religious and other organizations in particular), and (iii) average levels of community and political trust (aggregates of the trust scales).	contextual social capital	Physical health-long-term illness, health problem or handicap that limits daily activities or the work. Mental health was assessed emotional well-being. Self-rated health (including both physical & mental health).	Household income and political trust were particularly important predictors of long-term illness, but community social capital were mostly irrelevant in this instance The strongest predictors of fair/poor health were age and political trust, followed by income and community level variables were not significantly related to self-rated health.	Only the measure of depressive symptoms had variability that could be reasonably attributed to the community and a mere 2.1% of variability (ICC) could be attributed. The other two measures of health, i.e., the presence of a long-term illness and self-rated health status, were predicted by individual-level factors only.
**Studies in Western Europe**						
**Drukker, Kaplan, Feron & van Os, 2003 [113]**	A longitudinal cohort study of 7236 children and their families in the city of Maastricht 36 neighbourhoods, in the Netherlands. Level 1: Individual (micro) Level 2: Neighborhood (Meso).	Social capital was measured using two collective efficacy scales: informal social control, and social cohesion and trust.	'Aggregated' social capital (individual level responses aggregated to neighborhood level).	Children's general health and satisfaction and the mental health and behaviour	Social capital non-specifically associated children's general health and satisfaction. The mental health and behaviour dimensions were more specifically associated with degree of informal social control in the neighborhood.	Variance component for both levels and/or ICC was not reported.
**Mohan, Twigg, Barnard & Jones, 2005 [114]**	A follow-up study based on English sample of 7578 individuals followed from1984/85 to 2001 modelled individual and ecological data simultaneously and data come from the Health and Lifestyle Survey(HALS) Level 1: Individual (micro) Level 2: Electoral wards (Meso).	Used area measurer of social capital on a range of indicators (drawn from various surveys) such as- participation in voluntary activities (from GHS); political activity, social activity, election participation, altruistic activity etc (from BHPS); friendly community and 'community sprit' (from SHE).	'Aggregated' social capital (individual level responses aggregated to electoral wards level).	The probability of individual mortality.	Not found conclusive evidence in support of social capital as a contextual construct which has an influence on health.	Variance component for both levels and/or ICC was not reported.
**Studies in Scandinavia**						
**Lindström, Moghaddassi, & Merlo, 2004 [15]**	A cross sectional study data came from the public health survey in Malmö, 1994. A total of 3,602 individuals aged 20–80 years living in 75 Neighbourhoods were considered. Level 1: Individual (micro) Level 2: Neighborhood (Meso).	The social participation was used as a proxy for social capital at the individual level. Individual- social participation defined as how actively the person takes part in the activities of formal and informal groups as well as other activities in society during the past 12 months. Items were summed and were classified as having low social participation (score was three or less activities out of 13 items).	'Aggregated' social capital (individual level responses aggregated to neighborhood level).	The influence of neighbourhood and individual factors on self-reported health in the neighborhoods of city of Malmö, Sweden.	The neighborhood level social capital is associated with self-reported health.	The neighborhood variance in self-reported health was mainly influenced by individual factors with 0.0% ICC.
**Cross-country studies**						
**Drukker, Buka, Kaplan Mckenzie & van Os, 2005 [115]**	A cross-sectional study based on data from (1) the Project on Human Development in Chicago Neighborhoods (PHDCN), USA and (2) the Maastricht Quality of Life study (MQoL), the Netherlands. For the PHDCN, 874 census tracts were combined to create 343 "neighborhood clusters" (NCs) consisting of approximately 8000 inhabitants each. NC Maastricht consists of 36 residential neighborhoods, housing between 300 and 8500 inhabitants, and all these neighborhoods were selected for the MQoL. Both the PHDCN and the MQoL consisted of a family cohort study as well as a community survey. Level 1: Individual (micro) Level 2: Neighborhood cluster/residential neighborhood (Meso).	Subjective neighborhood social capital used and operationalized by perception about informal social control (ISC) and social cohesion and trust (SC&T) that developed by Sampson and colleagues [107] and construct scales consist of 5 items each and respondents answered these on a 5-point Likert scale.	'Aggregated' social capital (individual level responses aggregated to the neighborhood level).	Children's (age11–12) perceived health measured in 5-item Likert type scale.	Chicago had lower levels of SC&T while Maastricht had lower levels of ISC. Higher levels of ISC and SC&T were associated with higher levels of children's perceived health, in both Maastricht and the Chicago Hispanic sub-sample, but not in the Chicago non-Hispanic samples.	Variance component for both levels and/or ICC was not reported.

### Single level studies (individual and ecological)

#### Studies in North America (USA & Canada)

Studies of social capital and health in the USA have found an association between a variety of indicators (trust, reciprocity, group membership) and health outcomes (lower all-cause and cause-specific mortality and better self-rated health) [17,74-78]. Ecological studies in the USA have primarily been carried out at the state level, with one exception [75] involving neighborhoods in the city of Chicago. In studying the effects of social capital all these studies used 'aggregated' level social capital. For instance, using three US General Social Survey (GSS) measures of social capital (individuals responses on the items such as, social distrust, perceived lack of fairness and helpfulness of others), Kawachi *et al. *reported that each of the measures of 'aggregated' social capital was associated with income inequality and mortality in the USA states [16]. Wilkinson *et al. *found that social mistrust is closely associated with mortality and violent crime rates in 39 US states [74].

Another state level ecological analysis was conducted by Milyo and Mellor [77]. They considered both crude and age-adjusted mortality rates as health measures and both contextual and 'aggregated' social capital were measured by Robert Putnam's index of state social capital and by an index of social mistrust derived from responses to the General Social Survey. However, the study did not find significant associations between mortality and either income inequality, minority racial concentration, or social capital. They concluded that different age-adjustment methods can cause a change in the sign or statistical significance of the association between mortality and other socioeconomic factors.

In Canada, Veenstra's findings for Saskatchewan are also different from most of the US studies. Veenstra found that individual level social capital (overall civic participation, trust in government, trust in neighborhood, etc) was not significantly related to self-rated health among the general population but had a positive impact among the elderly [79]. In another study, he showed that individual level social capital (associational density and civic participation) was inversely and weakly associated with age-adjusted mortality rates but not with low-birth weight rate [80]. In a recent study, Veenstra *et al. *(2005) found that individual level social capital (measured by constructing an index focusing specifically on breadth and depth of involvement in voluntary associations) particularly, overall involvement in voluntary associations was significantly associated with emotional distress and body-mass index and marginally related with self-rated health before and after controlling for age, gender, and neighborhood of residence [81]. They concluded that more participation in voluntary associations apparently had a positive association with well-being operationalized by these health measures.

#### Studies in eastern Europe

In Russia, Kennedy *et al. *showed that 'aggregated' social capital indicators (social cohesion, civic engagement and trust in government) were strongly associated with lower mortality rates during the period of the Russian mortality crisis [8]. Rose found that both human capital and individual level social capital measures (sense of self-efficacy, trust of others, informal networks and social support) were associated with better self-rated health in Russia [82]. Skrabski and colleagues also found associations between 'aggregated' social capital variables (lack of social trust, reciprocity between citizens and help received from civil organizations) measured at the county level in Hungary and rates of middle age mortality and all cause mortality rates [83,84].

#### Studies in western Europe

Using the British Household Panel Study for the years 1998 and 1999, McCulloch found that lower individual level social capital (social disorganization) was associated with increased risk of psychiatric morbidity [85].

Considering vertical contextual social capital (party political affiliation and general election voting pattern was used to measure social capital) in Ireland, Kelleher *et al. *found that there was no significant relation between Standardized Mortality Ratios (SMR) and voting pattern for the two main political parties but a significant relation with left wing voting [86]. There was a significant positive relation between left wing voting and dissatisfaction with health as measured from selected reported measures of health status and lifestyle.

#### Studies in Scandinavia

In Finland, Hyyppä and Mäki compared active life expectancies and disability pensions between two neighboring regions consisting of both bilingual (half Swedish and half Finnish) and entirely Finnish-speaking municipalities [87]. They found that the individuals belonging to the Swedish speaking community remained active in working life longer and had a significantly longer life span than their Finnish speaking counterparts in the same region. They suggested that the observed inequalities in active life and in mortality depend on differences in the extent of individual level social capital. In another study Hyyppä and Mäki found that the Swedish speaking community seemed to have access to a greater stock of individual level social capital than their Finnish speaking counterparts, which in turn was significantly and positively associated with better self-rated health [88]. Using the same data set, Hyyppä and Mäki recently examined which social capital indicators were important for health and sought to identify possible pathways through which social capital might influence individual health [88]. After controlling for health-related behavior, the results showed that Finnish speakers were more likely to be migrants, to mistrust others, and to be less active in community events. They also concluded that active participation in voluntary associations and friendship ties are associated with better self-rated health in a bilingual community.

Using a prospective cohort study design among public sector employees in Finland, Liukkonen and colleagues found that a low level of individual level 'workplace social capital' constructed from information on employment security and social support/co-worker support and combining them into a variable indicating the amount of 'social job capital'/social capital was associated with poor health (measured by a 5-point scale of self-rated health and psychological distress) [90]. However, in the age-adjusted model this significant association remained only for women. After accounting for baseline health differences and other background variables, any significant associations for either women or men disappeared.

Employing a set of individual panel data from Statistics Sweden's Survey of Living Conditions (the ULF survey), Bolin *et al. *reported that individual level social capital (i.e. having a close friend outside of the household) had a positive effect on self-rated health [18]. Using a cross-sectional follow-up study based on data (from 1990 and 1991) from the Swedish Annual Level-of-Living Survey (SALLS), Sundquist *et al. *concluded that persons with low social participation in the social participation index had an increased risk of CHD [91].

More recently, employing cross-sectional data from Scania, the southernmost region of Sweden, Lindström constructed a social capital indicator. Based on both individual social participation in society and individual perception of generalised trust of other people combined with social participation and trust, the indicator yielded four alternative social capital categories: high-social participation/high trust (*high social capital*), high-social participation/low trust ("*miniaturized community*"), low-social participation/high trust (*traditionalism*), and low-social participation/low trust (*low social capital*) [92]. The author found that for both males and females low trust was significantly associated with higher probability of poor self-reported global health. The odds ratios of poor self-reported health were significantly higher in the categories of high-social participation/low trust ("*miniaturized community*"), low-social participation/high trust ("*traditionalism*") and low-social participation/low trust (low-social capital) compared to the high-social capital (high-social participation/high trust) category among both men and women. The highest probability of poor self-reported global health was observed in the low-social capital categories in both sexes.

#### Studies in Australia

Using state level data, collected from several Australian Bureau of Statistics documents for the years 1990–1996, and five different indicators of social integration (percentage of people living alone, divorce rate, unemployment rate, proportion of people who are discouraged job-seekers and unionization rate) as a proxy for contextual social capital, Siahpush and Singh found that higher levels of most of the indicators of social capital were significantly associated with lower state level different cause-specific mortality, all cause mortality and sex specific life expectancy in Australia [93].

However, employing household level cross-sectional data among two disadvantaged neighborhoods in south-western Sydney and using six common individual level social capital components (neighborhood attachment, support networks, feelings of trust and reciprocity, local engagement, personal attachment to the area, feelings about safety and pro-activity in the social context), Chavez *et al. *concluded that, with the exception of feelings of trust and reciprocity, no other social capital component made significant contributions to explain self-rated health variance and that macro-level factors such as housing conditions and employment opportunities emerged as key explanatory factors[94].

Similar results were also found by Ziersch *et al. *in the Western suburbs of Adelaide, Australia considering respondent's physical health as dependent variable and individual level social capital as operationalized by neighborhood connections, neighborhood trust, reciprocity, neighborhood safety, and local civic action [95]. They showed that only perceived neighborhood safety was related to physical health and neighborhood connections and neighborhood safety were positively associated with mental health, proving subjects with stronger neighborhood connections and higher levels of perceived neighborhood safety to have better mental health.

#### Cross-country studies

Using World Values Survey (1990–1991) data for 16 OECD countries, Lynch *et al. *observed weak and inconsistent associations between a country's 'aggregated' level of social capital (social distrust, organization membership and volunteering) and age-specific and cause specific mortality rates [96].

Employing panel data from 19 OECD countries, Kennelly and colleagues also found little statistically significant positive associations between 'aggregated' level social capital (general trust and membership in voluntary organization) and population health as measured by three indicators of health status – life expectancy at birth, infant mortality and perinatal mortality [97].

Using data from the World Values Survey, 1995–97, Smith and Polanyi also found that individual level social capital (socially oriented behaviors and existence of socially oriented norms or behaviors) did not reduce the likelihood of lower income groups reporting poor self-rated health, relative to the highest income groups [98].

Employing World Value Survey data (for the years 1995–97) for 18 European countries, another cross-country study was done by Carlson where social capital was measured from an individual perspective, conceptualized as the degree of the individual's trust in people, confidence in the legal system, or membership of organizations [99]. The study found that both economic factors and some aspects of individual level social capital play a role in the area differences in self-rated health. However, economic factors appeared to be more important. People in the countries in central and eastern Europe tend to be worse off than in western Europe, both in terms of economy and in terms of social capital.

Helliwell and Putnam used large samples of data from three different sources, namely the World Values Survey, a three-wave panel for the years 1980, 1991–1992 and 1995–1997 covering 49 countries, the Social Capital Benchmark Survey in the USA, and Canadian data drawn from two national waves and two special over-samples of a survey. They found that individual level social capital (measured by indicators such as the strength of family, neighborhood, religious and community ties) was strongly associated with subjective well-being through many independent channels and in several different forms. They concluded that all indicators of social capital appeared independently and robustly related to happiness and life satisfaction, both directly and through their impact on health [100].

A cross-national study based data on Germany and the USA was done by Pollack and Knesebeck, where individual level social capital indicators included both norms (reciprocity and civic trust) and behaviors (social participation) [101]. The study showed that lack of reciprocity was associated with poorer self-rated health in both countries. Civic mistrust was associated with poorer self-rated health in both countries and with functional limitations and depression in the USA but not in Germany. Lack of participation was found to be associated with poorer self-rated health and depression in Germany and the effect of norms was found to be stronger in the USA than in Germany.

### Multilevel studies

Multilevel regression analysis allows us to identify and quantify the extent to which variations in health are attributable to the characteristics of the area in which the individual resides. In particular, this technique enables us to determine if area level social capital affects individual health over and above, or in interaction with, individual characteristics. Multilevel regression analysis allows researchers to investigate how much of the area differences in health can be explained by differences in the individual composition of an area, and how much of these area differences are explained by their level of social capital. Among other reasons for applying multilevel regression techniques is (using statistical terminology) the existence of an area conditioned residual correlation. Failure to consider this dependence produces an underestimation of the standard errors of the regression coefficients [102,103].

Multilevel analysis, initially applied in the areas of sociology, education, and demography, has recently attracted increased attention in the field of public health [73]. Researchers are also bringing to light the role of contextual and environmental factors on health [104-106]. There are two complementary approaches to understanding contextual effects (either based on derived or integrated variables) on individual health [104]. One approach – the analysis of traditional measures of association or fixed effect results – focuses on understanding how area characteristics are associated to individual health over and above individual factors. The other approach – the analysis of measures of health variation or random effect results – focuses on how health outcomes are distributed within and between different levels. Both approaches deserve to be investigated since they provide relevant and complementary information. In the present paper we summarize both the fixed effect results and random effect results of published multi-level studies, as this is a key to understanding the interpretation made by the authors. Table 2b provides a summary of the recent multilevel studies on social capital and health.

#### Fixed effect results

##### Studies in North America (USA & Canada)

Multilevel studies based on data from the USA have generally demonstrated a significant fixed effect of area level social capital on population health. With the exception of one, all studies used 'aggregated' social capital at the area level [see, [107-111]]. After adjusting for individual compositional variations, these studies found that living in an area with higher level social capital (measured by the indicators such as civic trust, reciprocity and civic engagement, volunteering, mistrust, interpersonal trust degree of trust worthiness of neighbors, density of local networking, and social cohesion) were strongly associated (fixed effects) with individual well-being and health [7,14,107-111].

For instance, Sampson *et al. *observed that an index including mutual trust and social control (social capital/social cohesion) is significantly inversely associated with neighborhood violence and homicide rates for the neighborhoods in Chicago [107]. Employing the 2000 Social Capital Community Benchmark Survey, Subramanian *et al. *examined the compositional and contextual effects of social trust on individual self-rated health across 40 US communities [14]. After controlling for individual demographic and socioeconomic status variables, the authors found an association between community levels of social trust (derived from individual level responses) and self-rated health. However, this association disappeared after controlling for individual trust perception in the model, although a significant cross-level interaction effect was found between community and individual trust. That is, self-reported health was lowest among individuals who expressed low trust and lived in communities with higher levels of trust.

Employing information on premature mortality from heart disease, Franzini and Spears found that although individual level characteristics were major predictors of premature heart disease mortality, social context/contextual social capital (operationalized by homeownership, i.e. percent of owner-occupied housing units at the tract and county level, and the crime index, defined as number of serious crimes known to the police per 100,000 population at the county level) at the block-group, tract, and county level also played an important role in explaining years of life lost to heart disease [111]. The study concluded that block-group level wealth, tract level own group ethnic density, and county level social capital had significant effects on years of life lost to heart disease in Texas.

A recent cross-sectional multilevel study was conducted by Veenstra where self-rated health (including both physical and mental health) was considered as a response variable and social capital was measured at both individual and community level [112]. Individual-level social capital was operationalized through individuals' perceptions about social and political trust and participation in voluntary associations and also measured attributes of communities such as (a) the number of public spaces per capita (sports, recreational, casual and social, cultural, religious, school, and hall spaces in particular), (b) the number of voluntary organizations per capita (sports and athletics, community, minorities, arts and culture, business, political, health and social services, religious and other organizations in particular), and (c) average levels of community and political trust (aggregates of the trust scales). The study found that the strongest predictors of fair/poor health were age, political trust and income, while community level variables were not significantly related to self-rated health. The study further concluded that household income and individual level political trust were particularly important predictors of long-term illness, but contextual social capital (at the community level) was mostly irrelevant in this instance.

##### Studies in western Europe

Using a longitudinal cohort study of 7236 children and their families in the city of Maastricht, the Netherlands, Drukker *et al. *found that 'aggregated' level social capital within neighborhoods was non-specifically associated with children's general health and satisfaction [113]. The mental health and behavior dimensions were more specifically associated with the degree of informal social control in the neighborhood.

In a recent study employing the Health and Lifestyle Survey (HALS), a follow-up study based on an English sample (from 1984/85 to 2001) and using different 'aggregated' level social capital indicators, Mohan *et al. *find little support for social capital as an area influence on the probability of survival [114].

##### Studies in Scandinavia

A significant fixed effect of social capital on self-rated health has also been observed in a Swedish study conducted by Lindström and colleagues. They assessed the influence of neighborhood level social participation and individual factors on self-reported health in the city of Malmö and concluded that 'aggregated' level social capital is significantly associated with individual self-reported health [15].

##### Cross-country studies

A recent study by Drukker *et al. *used data from the Project on Human Development in Chicago Neighborhoods (PHDCN), USA and the Maastricht Quality of Life study (MQoL) [115]. Subjective neighborhood social capital was used and operationalized through individual perceptions about informal social control (ISC) and social cohesion and trust (SC&T). Children's (age 11–12) perceived health was measured using a 5-item Likert type scale. Their findings indicated that Chicago had lower levels of SC&T while Maastricht had lower levels of ISC. The differences in both ISC and SC&T between the two cities are approximately half a standard deviation. Moreover, higher levels of 'aggregated' ISC and 'aggregated' SC&T were associated with higher levels of children's perceived health, in both Maastricht and the Chicago Hispanic sub-sample, but not in the Chicago non-Hispanic samples.

#### Random effect results

In investigating contextual/area determinants of health, the multilevel modelling approach allows for partitioning of variation arising at different levels of the hierarchy (e.g. individual and area), and explicit modelling of this variation allows for such insights to be made [116]. To assess the extent to which contextual effects of areas play a role in determining individual health or health behavior, the variance partition coefficient (VPC) or intra-cluster correlation (ICC) statistics provides an approach for identifying and quantifying area influences on population health. However, it is evident from our review that most studies do not report random part results for both higher and lower level variances, nor do they typically report ICCs.

##### Studies in North America (USA & Canada)

Random part results not being reported in their entirety (or ICCs) is rather common for the USA studies. However, the report by Sampson *et al. *from the Project on Human Development in Chicago Neighborhoods (PHDCN) is one exception [107]. In examining the relationship between neighborhood level collective efficacy and violent crime they reported both variance components for within neighborhoods (0.320) and between neighborhoods (0.026) and the ICC was estimated about 7.51%.

In the Canadian context Veenstra also reported both variance components. He found that two measures of health, i.e. the presence of long-term illness and self-rated health status, were predicted by individual-level factors only (i.e. with 0% ICC). The measure of depressive symptoms had some variability, (with 2.1% ICC) that could be reasonably attributed to the community [112].

##### Studies in western Europe

The studies included in this review did not report random part results.

##### Studies in Scandinavia

In contrast to the North American studies, area level random parameters have generally been found to be very low (low ICC) in Swedish studies. Lindström and colleagues observed that neighborhood variance in self-rated health was mainly affected by individual factors other than individual social capital (assessed by social participation) with 0.0% ICC [15].

##### Cross-country studies

The studies included in this review did not report random part results.

### Summary of findings

We have reviewed 30 single level studies (using both individual and ecological level data) and 12 multilevel studies from different countries, mainly OECD countries in North America, Europe (both eastern and western) and Australia. It is evident that most of the studies operationalized social capital as a combination of both cognitive (particularly, trust and reciprocity) and structural (informal participation or civic engagement) dimensions of social capital. Moreover, to construct the area level social capital variable most of the studies used the 'aggregated' type of social capital. Out of 42 studies, four studies employed both an 'aggregated' and a contextual social capital measure and four studies (2 single level and 2 multilevel) employed merely contextual social capital in analyzing the health impact of contextual effects.

A summary of results on the associations between social capital and health by different countries is presented in Table 1. Studies using single level analysis found significant relationships between social capital and health. Particularly for the USA virtually all studies found a strong association between lower mortality/better health status and greater stocks of social capital. For Canada, the findings were weaker and less consistent. Except for one Finnish study, significant associations were also observed in both eastern and western Europe including the Scandinavian countries. The findings from Australia were mixed. Two studies reported fairly weak associations between social capital and health. Studies based on cross-country and World Values Survey Data also showed very little or weak and inconsistent associations between social capital and self-rated health or mortality.

For multilevel studies, irrespective of the country's overall degree of material egalitarianism, the fixed-effect results found significant relationships between health and social capital. However, when looking at the studies' random part results one finds that they reported dissimilar results depending on the country's context. In the context of societies with higher degrees of egalitarianism (countries such as Sweden and Canada), multilevel studies report very small ICCs for health outcomes. By contrast, for countries which are not egalitarian, for instance the USA studies reveal higher ICCs, implying greater neighborhood variance in mortality or health. One interpretation is that neighborhoods or areas (characterized by stock of social capital) play a negligible role in explaining health variations in egalitarian countries, even if it was found to be significantly associated with health. In other words, area characteristics such as social capital appear to play a comparatively greater role in less egalitarian societies.

## Discussion and conclusions

In this review we have tried to provide an overview of the definitions and forms of social capital and the probable theoretical associations between social capital and health. Recent empirical studies on associations between individual and area level social capital with various health outcomes have also been reviewed systematically. To summarize, it is evident from our review that most of the studies consider some combination of cognitive and structural forms of social capital and operationalize social capital using individual level trust and reciprocity along with informal participation as indicators. Only one study considered the vertical dimension of contextual social capital using Irish data. Although different studies have conceptualized social capital and combined its several components rather differently, irrespective of a country's overall degree of economic egalitarianism, most single level studies have reported a positive association between social capital (both at the individual level and area level) and better health. However, the findings seemed different for studies employing multilevel analysis where the health impacts of 'aggregated'/contextual social capital have been separated from individual compositional effects. Multilevel studies based on US data have generally demonstrated area level fixed and random effects of social capital on population health with higher intra-cluster correlation. By contrast, Swedish and Canadian studies supports an association in the fixed effects, but area level random parameters are generally very low (i.e. low ICC).

Due to the small number of multilevel studies and very few studies reporting random part results, it is difficult to draw any definite conclusions on the basis of our observations. Nonetheless, the proportion of area level variance to total variance (ICC) was quantitatively similar to those presented in the literature emphasizing the significance of area effects. For instance, Fisher *et al. *examined the variation in self-reported physical activity among older adults in Portland, USA and concluded that social capital (social cohesion) is associated with increased levels of physical activity among older adults with an estimated ICC of 4% [117]. Using British household cross-sectional data, McCulloch tested variations in neighborhood social capital and social disorganization. For social capital he found ICCs of 9.0% for men and 11.3% for women, while for social disorganization the estimated ICCs were even higher, 21.2% for men and 25.5% for women [118]. The Scottish Heart Health Study found that ICC (district level variance of total variance) was between 0.5% (serum cholesterol) and 5.7% (alcohol consumption and smoking) for coronary heart disease risk factors [119].

On the other hand, in a recent study using a four-year follow up study of the entire Swedish population aged 40–64, Sundquist *et al. *examined whether neighborhood deprivation (another area attribute, an index measured at small area market statistics level by the use of Care Need Index) predicts incidence rates of coronary heart disease (CHD) [120]. They concluded that high levels of neighborhood deprivation independently influence CHD for both men and women, although the ICCs were reported at 0.9 % for men and 2.1% for women. Other Swedish studies also reported similarly low ICCs. For instance, Lindström and colleagues observed that neighborhood variance in daily tobacco smoking was mainly affected by individual factors other than individual social capital (assessed by social participation) and the intra-neighborhood correlation was 1.9% (ICC) [121]. In another study, Lindström *et al. *concluded that leisure time physical activity is mainly influenced by individual factors, and area level social capital (aggregated) did not explain the neighborhood difference in physical inactivity. They reported the ICC (intra-neighborhood correlation) to be 2.1% [122].

Some caveats may be identified from our review. Firstly, as we have observed, different studies conceptualized social capital differently within the same country and/or across the countries. How robust are these observations considering the fact that different researchers conceptualize social capital differently? In other words, one could raise the question if we are indeed comparing the same phenomenon across the countries? In particular, social participation and trust have been suggested in the literature as representing different aspects of social capital and both these aspects of social capital are thought to mutually enhance each other [123]. However, in reality this is not always the case. According to Fukuyama's notion of 'the miniaturization of community', the more ideologically narrowly defined social networks and organizations destroy generalized trust between people and this phenomenon is observed in the USA [124]. In the Swedish context, Lindström also makes it evident that poor self-reported health is significantly more prevalent in miniaturized (high social participation/low trust) communities compared to high social capital (high social participation/high trust) communities [92]. However, because of limited numbers of studies across countries, the study found difficulties in further disaggregating studies with similar social capital indicators or health outcomes.

The second issue is a corollary of the first; because of small numbers of studies it is difficult to draw definitive conclusions on the basis of our observations. Moreover, social capital may not always generate better health outcomes. Because of the potential importance of social capital externalities (positive and/or negative), the path from individual to aggregate social capital may be difficult to identify [3]. The benefits that social capital creates for one group may disadvantage another, so that the net spill-over effect on society need not be positive [43]. It was also not possible to scrutinize intra-group effect of social capital through our review.

Finally, most of the studies included in our review had a number of methodological limitations. We may exemplify some of the statistical issues that could be regarded as important here. Firstly, for cross-sectional studies (which is the basis of most of the studies), one cannot exclude the possibility of reverse causality. It is recognized that in many contexts, social capital is endogenous and studies have not employed instrumental variables to allow for consistent estimation of parameters. This problem may be expected to be more problematic for individual level social capital studies. In addition, due to imprecision in the indicators of area level social capital executed in different studies, there is the possibility of measurement errors in the explanatory variable and any such errors would lead to inconsistent estimation of models [125]. A general limitation is failure to include other relevant area level predictors in the models. This could mis-specify the fixed part of a multilevel-level statistical model. However, researchers should be cautious about including other contextual predictors and keep in mind the multicolinearity problem. Keeping these methodological limitations in mind, the conclusions observed in some of the reviewed studies need to be interpreted with caution.

## Conclusion

It is evident from the fixed effect results across studies (both single level and multilevel) and countries that the positive health impact of social capital and a country's degree of egalitarianism seemed rather unimportant factors in modifying the effects of social capital on health. Secondly, although a significant fixed effect association was observed between area level social capital and better health in multilevel studies, low variability in health across areas reported in some studies suggests that the differences in health were predominantly affected by individual factors rather than by area characteristics, especially in egalitarian countries.

We have raised the question whether egalitarianism matters in mediating the health impact of social capital and have tried to find some answers through reviewing existing literature. However, many questions still remain unanswered about the notion of social capital and interrelations between social capital and health. Before making further definitive conclusions that social capital is an important determinant of population health (or health differences) or that the egalitarian distribution of income and wealth matters for conditioning the health impact or differences of social capital, careful exploration is warranted through employing appropriate statistical methods (e.g. multilevel analysis) and data. It is expected that the observations from this review will have implications for the content and direction of future research concerning social capital and social determinants of health, on policy formation as well as on the implementation of health service delivery.

## Authors' contributions

M. K. Islam, U-G. Gerdtham and J. Merlo conceived the study. M. K. Islam, U-G. Gerdtham, J. Merlo, I. Kawachi and M. Lindström designed the study. M. K. Islam analyzed and wrote the paper. All authors provided significant comments, wrote, read, and approved the final manuscript.
